# Implementing Findable, Accessible, Interoperable, Reusable (FAIR) Principles in Child and Adolescent Mental Health Research: Mixed Methods Approach

**DOI:** 10.2196/59113

**Published:** 2024-12-19

**Authors:** Rowdy de Groot, Frank van der Graaff, Daniël van der Doelen, Michiel Luijten, Ronald De Meyer, Hekmat Alrouh, Hedy van Oers, Jacintha Tieskens, Josjan Zijlmans, Meike Bartels, Arne Popma, Nicolette de Keizer, Ronald Cornet, Tinca J C Polderman

**Affiliations:** 1 Department of Medical Informatics, Amsterdam University Medical Center, University of Amsterdam, Meibergdreef 9, Amsterdam, 1105 AZ, Netherlands, 31 648499049; 2 Amsterdam Public Health, Digital Health, Amsterdam University Medical Center, Amsterdam, Netherlands; 3 Praktikon, Nijmegen, Netherlands; 4 Karakter Child and Adolescent Psychiatry University Centre, Nijmegen, Netherlands; 5 Emma Children’s Hospital, Child and Adolescent Psychiatry & Psychosocial Care, Amsterdam University Medical Center, University of Amsterdam, Amsterdam, Netherlands; 6 Amsterdam University Medical Center, Vrije Universiteit Amsterdam, Epidemiology and Data Science, Amsterdam, Netherlands; 7 Amsterdam Public Health, Mental Health, Amsterdam University Medical Center, Amsterdam, Netherlands; 8 Department of Biological Psychology, Vrije Universiteit Amsterdam, Amsterdam, Netherlands; 9 Leiden University Medical Center Curium–Child and Adolescent Psychiatry, Leiden University Medical Center, Leiden, Netherlands; 10 Department of Child and Adolescent Psychiatry & Psychosocial Care, Amsterdam University Medical Center, Vrije Universiteit Amsterdam, Amsterdam, Netherlands; 11 Levvel, Academic Center for Child and Adolescent Psychiatry, Amsterdam, Netherlands; 12 Department of Child and Adolescent Psychiatry, University Medical Center Groningen, University of Groningen, Groningen, Netherlands

**Keywords:** FAIR data, research data management, data interoperability, data standardization, OMOP CDM, implementation, health data, data quality, FAIR principles

## Abstract

**Background:**

The FAIR (Findable, Accessible, Interoperable, Reusable) data principles are a guideline to improve the reusability of data. However, properly implementing these principles is challenging due to a wide range of barriers.

**Objectives:**

To further the field of FAIR data, this study aimed to systematically identify barriers regarding implementing the FAIR principles in the area of child and adolescent mental health research, define the most challenging barriers, and provide recommendations for these barriers.

**Methods:**

Three sources were used as input to identify barriers: (1) evaluation of the implementation process of the Observational Medical Outcomes Partnership Common Data Model by 3 data managers; (2) interviews with experts on mental health research, reusable health data, and data quality; and (3) a rapid literature review. All barriers were categorized according to type as described previously, the affected FAIR principle, a category to add detail about the origin of the barrier, and whether a barrier was mental health specific. The barriers were assessed and ranked on impact with the data managers using the Delphi method.

**Results:**

Thirteen barriers were identified by the data managers, 7 were identified by the experts, and 30 barriers were extracted from the literature. This resulted in 45 unique barriers. The characteristics that were most assigned to the barriers were, respectively, external type (n=32/45; eg, organizational policy preventing the use of required software), tooling category (n=19/45; ie, software and databases), all FAIR principles (n=15/45), and not mental health specific (n=43/45). Consensus on ranking the scores of the barriers was reached after 2 rounds of the Delphi method. The most important recommendations to overcome the barriers are adding a FAIR data steward to the research team, accessible step-by-step guides, and ensuring sustainable funding for the implementation and long-term use of FAIR data.

**Conclusions:**

By systematically listing these barriers and providing recommendations, we intend to enhance the awareness of researchers and grant providers that making data FAIR demands specific expertise, available tooling, and proper investments.

## Introduction

The FAIR (Findable, Accessible, Interoperable, Reusable) data principles [1], published in 2016, are a guideline to improve the reusability of health data. These principles imply, respectively, the following: (1) Findable—metadata (ie, data about the data) and data should be easy to find for both humans and computers; (2) Accessible—the user needs to know how data and metadata can be accessed, possibly including authentication and authorization; (3) Interoperable—the data usually need to be integrated with other data; the data need to interoperate with applications or workflows for analysis, storage, and processing; and (4) Reusable—metadata and data should be well described so that they can be replicated and combined in different settings [2].

The implementation of the FAIR principles and thereby the increase of availability and usability of data advances scientific knowledge in several ways. First, it benefits researchers who have no sources for data collection themselves, for example, from low- and middle-income countries. Second, it enhances data sharing across cohorts, registers, or study samples worldwide, thus enlarging the scale, statistical power, and impact of empirical studies. Finally, it invites researchers from different disciplines to provide their unique perspectives and expertise on the exchanged data. In short, FAIR data maximize the usability of carefully collected, valuable data. In addition, FAIR data can contribute to a clear overview of which data are available for specific research questions (and as such avoid spending resources on collecting similar data) and facilitate the potential replication of published research, all adding to the reliability, validity, and value of scientific knowledge in general. Researchers increasingly aim to implement the FAIR principles [3]. In a study by Kersloot et al [4], after reading a short description of the FAIR principles, scientists were asked how much effort they spent on making their data more FAIR. About 80% indicated that they have spent effort to make their data more FAIR, addressing 1 or more of the 4 aspects [4].

However, despite a positive attitude of researchers regarding the FAIR principles, it appears challenging to properly implement them [5,6]. The FAIR principles provide guidance on *what* to do but not on *how* to do it. Implementing the FAIR principles requires expert knowledge of data, metadata, identifiers, ontologies, and terminologies, but there is a lack of guidelines and tooling, leaving it to researchers to assess which technology or resources should be used to achieve FAIRness.

For health data, it might be even more difficult to implement the FAIR principles, since one of the considerable limitations is privacy regulation, such as the European Union’s General Data Protection Regulation (GDPR). This complicates the “A”—accessibility aspect of FAIR, as more complex access procedures need to be described due to required security measurements. Of note, FAIR is often confused with open data; however, this is not the same. FAIR aims to facilitate federation, which deals (at least in part) with privacy matters and does not aim to make data freely available to everyone.

This study focuses on the FAIRification of mental health data. More than 13% of people worldwide suffer from mental illness [7], which has a major impact on global health. Thus, optimizing knowledge development in this health domain is crucial [8,9]. However, Sadeh et al [9] conclude that the mental health domain has not yet developed a culture around FAIR data or required tools and resources to easily FAIRify mental health data. We aim to identify the concrete barriers to FAIRification in the domain of mental health data and provide recommendations for these barriers to move the field of FAIR data in general and specifically for mental health further.

## Methods

### Overview

We used three sources as input to identify barriers: (1) evaluation of the implementation process of the Observational Medical Outcomes Partnership (OMOP) Common Data Model (CDM) by 3 data managers at 3 research sites; (2) interviews with experts in mental health research, reusable health data, and data quality; and (3) a rapid literature review. The research was directed by a qualified data steward (RdG), who categorized the identified barriers. Using the Delphi method [10], the 3 data managers scored the impact of the barriers on the FAIRification process. Subsequently, the top 10 most impactful barriers were determined. Recommendations for these barriers were derived from the authors and relevant scientific literature based on the rapid review.

### OMOP CDM

OMOP CDM is an open community data standard maintained by the OHDSI (Observational Health Data Sciences and Informatics) community that is used to represent observational data in a standardized manner. This standard ensures both semantic interoperability, where data are understood across systems, and syntactic interoperability, where the format of data is usable across systems. This allows researchers to write programs or analyses that work on multiple OMOP CDM instances. It also allows researchers to combine data, although OMOP CDM is more focused on federated analyses (ie, researcher A writes a script that researcher B can run on their OMOP CDM instance and researcher B sends back the results, thus without sharing data). OMOP CDM makes it also possible to use the OHDSI research tools and to perform collaborative research or large-scale analytics [11].

A data standard to increase the interoperability of the collected data was required. Therefore, together with the data managers we chose to implement OMOP CDM since it is oriented toward health-related observational data and makes the data more interoperable (the I of FAIR).

### Research Sites

The 3 research sites that participated in implementing OMOP CDM were KLIK [12], DREAMS (Dutch REsearch in child and Adolescent Mental health), and the Learning Database Youth (in Dutch: LDJ) [13,14]. These 3 research sites, among other research sites, were involved in a consortium to assess the mental health problems in Dutch children and adolescents before and during the COVID-19 pandemic [15,16]. These sites used a variety of questionnaires to collect data on mental health and demographic variables, such as sex, age, and socioeconomic status, from children, adolescents, and their parents with and with no preexisting mental health problems. These 3 research sites were part of a national consortium that collaborated on research on mental health of children and adolescents during the COVID-19 pandemic and concerned all sites that intended to harmonize data in the participating research sites.

KLIK is established in an academic hospital and collects data through a dedicated research website for research and to monitor children. DREAMS is a collaboration between 4 academic child and adolescent psychiatry centers that collects data for research and aims to improve mental health care for children. LDJ is a cooperation between more than 30 youth care institutions in the Netherlands that collects data on the mental health of children and adolescents receiving youth care to improve the quality of care. An overview of the research sites and their data represented in OMOP is shown in Table 1. Each of the 3 research sites had a data manager that was tasked to implement OMOP CDM (DvdD, FvdG, and ML). The 3 data managers were supported by a data steward experienced in implementing OMOP CDM (RdG). None of the research sites or data managers had previous experience with FAIR data or the implementation of OMOP CDM. Two of the 3 data managers had no experience with Structured Query Language, a standardized programming language that is used to manage relational databases and is necessary for the implementation of OMOP CDM.

**Table 1.** Overview of research sites and datasets (the Cantrill Ladder score is used to measure well-being).

	KLIK	DREAMS^a^	LDJ^b^
Description	Developed in an academic hospital and collects data through a research website developed for collecting data	A collaboration between 4 academic child and adolescent psychiatry centers, collects data for research, and aims to improve mental health care for children	A cooperation between more than 30 youth care institutions in the Netherlands that collects data on the mental health of children and adolescents receiving youth care to improve the quality of care
Data subset implemented in OMOP^c^	Person ID, age, sex, Cantrill Ladder score, PSQ^d^, and PROMIS^e^ anxiety	Person ID, sex, Cantrill Ladder score, PSQ, PROMIS anxiety, and date of questionnaire	Person ID, date of birth, sex, PSQ, and date of questionnaire

a DREAMS: Dutch REsearch in child and Adolescent Mental health.

b LDJ: Learning Database Youth.

c OMOP CDM: Observational Medical Outcomes Partnership Common Data Model.

d PSQ: Parenting Stress Questionnaire.

e PROMIS: patient-reported outcomes measurement information system.

### Barriers

In this paper, we define a barrier as an impediment to the implementation of the FAIR data principle. Usually, a barrier requires more resources, people, funding, or time to solve. For example, the GDPR does not prevent the implementation of the FAIR principles but requires more knowledge on what is and is not allowed. This could lead to the need for technical measures, for example, to prevent unrestricted access to the data.

**Figure 1.**
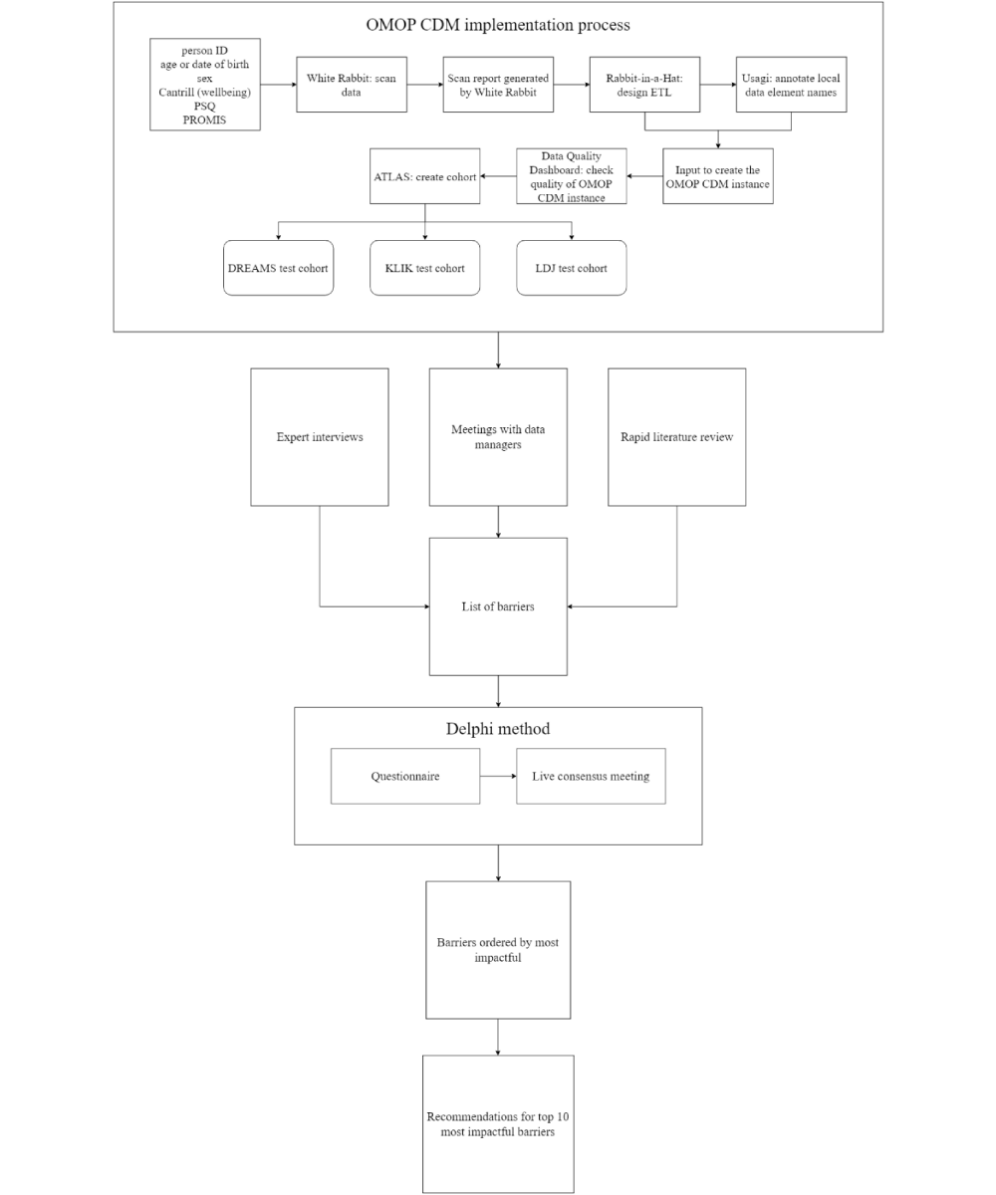
Overview of the process of the study. The entire process was coordinated by the data steward (RdG). Tooling: ATLAS is a tool to create cohorts in OMOP CDM; Cantrill Ladder score is used to measure well-being; Data Quality Dashboard is used to assess the quality of the data in OMOP CDM; Rabbit-in-a-Hat is a tool to design the conversion to OMOP CDM; Usagi is a tool to annotate local terms to standard concepts in OMOP CDM; White Rabbit is a tool to scan data. DREAMS: Dutch REsearch in child and Adolescent Mental health; ETL: Extract Transform Load; LDJ: Learning Database Youth; OMOP CDM: Observational Medical Outcomes Partnership Common Data Model; PROMIS: patient-reported outcomes measurement information system; PSQ: Parenting Stress Questionnaire.

First, barriers were identified by 3 Dutch data managers (FvdG, DvdD, and ML) during the implementation of OMOP CDM [11] for data from 3 mental health research sites, as described in the “Research sites” section. Second, we also collected barriers mentioned during interviews with a team of experts on reusable health data (RC), child and adolescent behavioral data (TJCP), and data quality (NdK). Third, we searched the scientific literature for relevant studies on implementing the FAIR principles and potential barriers. The process of collecting barriers and determining the results is shown in Figure 1.

### OMOP CDM Implementation

Since none of the data managers had previous experience with OMOP CDM, and the research centers collected partly different data elements, we started by modeling in OMOP CDM a small subset of the research data of each site. The subset consisted of a person ID, age or date of birth, sex, a Cantrill Ladder assessment of well-being [17], a Parenting Stress Questionnaire (PSQ) [18], a PROMIS (patient-reported outcomes measurement information system) questionnaire assessing anxiety [19], and the dates of filling in the questionnaires. For the DREAMS and KLIK research sites all data items were available while the LDJ research site did not collect the Cantrill Ladder assessment and the PROMIS anxiety questionnaire. Representative synthetic data were used in the OMOP CDM implementations.

We followed standard OHDSI steps for the OMOP CDM implementation process [20]. The OMOP process started by analyzing the data with White Rabbit. White Rabbit is a tool provided by OHDSI to scan datasets to provide information on tables, fields, and values. White Rabbit generates a scan report that is necessary to use Rabbit-in-a-Hat. OHDSI provides Rabbit-in-a-Hat to help users design their Extract, Transform, Load (ETL) process to represent their data in OMOP CDM. Usagi was used by the DREAMS and KLIK research sites to annotate local data element names to standardized concepts from the OMOP vocabulary for the Cantrill Ladder assessment and the PROMIS pediatric anxiety. The next step was to implement the ETL in a database and check the quality of the ETL with the data quality dashboard [21]. The last step was to use Atlas, a tool to conduct (research) analyses on OMOP CDM data [22], to construct a cohort and show that analyses can be performed on the converted OMOP CDM data. During the implementation process, 15 designated meetings were planned in which the data steward discussed the progress of the OMOP process and the encountered barriers with the data managers.

### Expert Interviews

The data steward (RdG) had expert interviews with 3 experts involved in the research from 3 different perspectives.

1. RC is a professor of medical informatics, focusing on semantic interoperability, both from a technical perspective and from a users’ point of view, as a key component to establishing FAIR data for health care and research.
2. TJCP is an associate professor of psychology and a research expert on child and adolescent behavioral data.
3. NdK is a professor of medical informatics, specializing in evaluating health care and health care information systems.

The interviews were held during 2 designated meetings in which the barriers identified by the data managers were reviewed by the experts. The experts together with the data steward (RdG) reflected on the barriers and complemented these with specific barriers from the experts. One additional meeting was held with a group of experts in child and adolescent mental health care during which the barriers from the data managers and experts were discussed.

### Literature

A rapid literature review was performed to identify barriers that hamper the implementation of the FAIR principles to both confirm and complement those encountered during the OMOP process or discussed during the expert interviews. The search term was “FAIR data implementation” in Google Scholar. No explicit inclusion or exclusion criteria were defined. If the title and abstract mentioned FAIR implementation barriers, we extracted challenges and barriers from the full text. We focused on FAIR data implementations in general to include as many papers as possible to make sure that we included a wide range of barriers. We searched only in Google Scholar since Google Scholar also encompasses results from other search engines such as PubMed.

### Categorizing the Barriers

All barriers to implementing the FAIR principles were categorized by the data steward. The first categorization was the type of barrier as described by Cabana et al [23]. The types of barriers were “Lack of Awareness,” “Lack of Familiarity,” “Lack of Agreement,” “Lack of Self-efficacy,” “Lack of Outcome Expectancy,” “Inertia of Previous Practice,” and “External Barriers.” Each barrier could be of 1 or more types of barriers. The second categorization regarded the FAIR principles or aspects of FAIR that were hampered due to the barrier. To further detail the origin of the barrier, a third categorization (the category) was added in which we specified whether people (skills or knowledge), tools, material, process, or costs were involved. Finally, we specified whether or not barriers were specific to the mental health care domain.

### Modified Delphi Method

We conducted a modified Delphi method as defined by Boulkedid et al [10] to establish consensus on the most impeding barriers to implementing the FAIR principles. First, a questionnaire including all identified barriers was sent to the 3 data managers to ask them to independently rate the barriers on a 1-to-10 scale on how much a barrier would hinder the implementation of the FAIR principles if it was encountered. The scores were totaled to determine the final score. The second round was a web-based meeting where the results were discussed with the supervision of the data steward. Barriers that had a scale score difference of 4 or more between any of the data managers were discussed, and clarified by the data steward, and scores were adjusted with justification if necessary. We considered a difference of 3 or fewer to reflect that each barrier could have a different experienced impact but still close enough for consensus. Justification of using the Delphi method is shown in Multimedia Appendix 1 according to the Guidance on Conducting and REporting DElphi Studies (CREDES) by Jünger et al [24]. We calculated percent agreement as a simple index for agreement between the 3 data managers and we calculated Krippendorff α as interrater reliability index [25]. Krippendorff α values range from 0 to 1, where 0 is perfect disagreement and 1 is perfect agreement. An α value higher than .8 is recommended whereas .667 is the lowest conceivable limit [26].

### Recommendations to Overcome the Barriers

Based on the results of the modified Delphi, we determined the top 10 most problematic barriers to focus on the most impactful barriers. We chose a top 10 list to provide a compact set of detailed recommendations, rather than a long list that would make it difficult to prioritize. In addition, some barriers, such as standardizing answers in open questions, cannot be solved. We believe that addressing the top 10 most problematic barriers will also help resolve many other issues, thus avoiding repetition. The barriers that scored highest were further examined to assess whether recommendations for overcoming these barriers were reported in the literature or could be determined or devised by the authors (RC and RdG, both experienced with FAIR data). We decided not to rank the recommendations, as they were virtually matched to all identified barriers. By ranking the barriers, we indirectly ranked the recommendations as well.

### Ethical Considerations

This study did not involve medical research and did not regard any interventions; no ethics approval was applied for in this study in line with the ethics approval procedures described on the Amsterdam Universitair Medische Centra website [27].

## Results

### Barriers

In total, 45 unique barriers to implementing the FAIR principles were identified. Of these, 13 barriers were identified during the OMOP CDM implementation process, 7 barriers were identified by experts, and 30 barriers were found in literature [6,28-36]. Five barriers were identified by all 3 sources. Multimedia Appendix 2 shows all identified barriers and their sources.

### Categorizing the Barriers

The “External barrier” type was assigned to most barriers: 71% (32/45). External barriers are barriers that hamper a researcher from implementing the FAIR principles, for example, organizational policy preventing the use of required software. Many barriers affected all of the FAIR principles (15/45, 33%). Interoperability and reusability principles were the most affected individual FAIR data aspects by 18% (8/45) and 20% (9/45) barriers, respectively. Principle I2: “(Meta)data use vocabularies that follow the FAIR principle” was the most affected FAIR principle with 9% (4/45) barriers, for example, answers to open questions in questionnaires cannot be standardized due to all answer possibilities. Approximately 42% (19/45) barriers were categorized in the “tools” category which was the most prevalent category, for example, updates can break software dependencies. Only 4% (2/45) of barriers were specific to mental health, namely (1) mental health data are collected in care modules (subcare pathways) that contain only questionnaires, and (2) the *DSM* (*Diagnostic and Statistical Manual of Mental Disorders*, ie, one of the core diagnostic instruments in mental health) does not make use of identifiers. Identifiers are necessary to refer to relevant metadata of the diagnosis. Approximately 11% (5/45) of barriers are more likely to occur in the mental health domain, mostly due to the extensive use of questionnaires, yet not exclusively applicable to mental health. All categorizations and scores of the barriers are shown in Multimedia Appendix 2.

### Modified Delphi Method

One data manager required more information from the data steward regarding certain barriers to score these. For 20 out of 45 barriers, consensus was reached by the data managers after filling in the questionnaire. The data managers reviewed the barriers for which there was no initial consensus, that is, a difference of 4 points or more between ratings. During the subsequent first live meeting, the 3 data managers discussed the 25 barriers without consensus after the questionnaire. During this meeting, for 21 out of 25 barriers the score was changed, resulting in a consensus for 19 barriers, while 6 barriers remained without reaching a consensus. The 6 barriers without consensus were again discussed during the second live consensus meeting in which the data steward participated in the discussion and consensus was reached for all barriers. The percentage of agreement was 55.6% before the first live consensus meeting, 86.7% after the first live consensus meeting, and 100% after the second live consensus meeting. Krippendorff α values were 0.34 (95% CI 0.15-0.52), 0.67 (95% CI 0.47-0.81), and 0.79 (95% CI 0.70-0.85), respectively, of which only the last value was close to the recommended value of 0.8. The lowest scoring barrier (answers to open questions in questionnaires cannot be standardized) had a score of 4 and the highest scoring barrier (Lack of technical knowledge or access to people with the necessary knowledge) had a maximum score of 30. All notes, scores, and comments of each round are shown in Multimedia Appendix 3.

### Recommendations to Overcome the Barriers

The recommendations for the top 10 barriers were derived from authors RC and RdG and the literature [37-42]. Table 2 shows the top 10 barriers and the recommendations to overcome these barriers. The main recommendations are to involve FAIR data stewards, create step-by-step guides for researchers to make their data FAIR, and implement sustainable funding that can maintain and support the FAIR data infrastructure, once it has been set up for a certain project.

**Table 2.** The top 10 impeding barriers of implementing the FAIR^a^ principles and recommendations to solve these problems.

Number and score	Top 10 barriers	Recommendation
#1 (30 points)	Lack of technical knowledge (of programs, languages, and error messages) or access to people with the necessary knowledge.	(1a) Researchers need a step-by-step guide to FAIRify their data that is also easy to understand with little technical knowledge. This guide should also include a section to make resources reusable by computers. This guide does not need to be useable by everyone but could apply to a specific community or a single organization. (1b) A checklist of required tools and software might provide useful as well and could be combined with teaching handbooks such as the handbook written by Engelhardt et al [39]). Many barriers we identified are related to tooling. Tooling and proper documentation could potentially help out in overcoming a lack of technical knowledge. (1c) Another approach is involvement of a (FAIR) data steward who will support the researchers in correctly implementing the FAIR principles (Scholtens et al [37]; Wendelborn et al 2023 [38]).
#2 (28 points)	The infrastructure to host FAIR data must remain active/online after the project or research is stopped.	Research funders need to provide a funding structure by which costs of making data FAIR and maintaining FAIR data infrastructure are covered, including the period after the project has ended or alternatives such as public or institutional repositories need to be considered to cover these costs.
#3 (28 points)	Organizational policies prevent researchers from implementing FAIR principles. Organizational support is required for setting up the infrastructure, including installing required software and databases.	Organizations need to have a streamlined process for their researchers to make their data FAIR and make sure that necessary tools and software are available for researchers (Jacobsen et al [40], FAIRification workflow, FAIR cookbook). See #1a. and #1b. for recommendations.
#4 (27 points)	Updates can break software dependencies.	Researchers can consider software that enables use of containerized applications such as Docker, Podman, or Buildah. However, the ability to implement software like this might depend on other barriers, such as technical skills or organizational policies that may prevent the implementation of certain software.
#5 (26 points)	Communication between different stakeholders that have different backgrounds is a challenge in a FAIRification process. A FAIR data steward seems essential to manage the necessary communication between disciplines (Queralt et al [6]).	Scholtens et al [37] argue that a (FAIR) data steward is vital for the communication between different disciplines and professionals (Scholtens et al [37]). We recommend that funding bodies or research-performing organizations have (FAIR) data stewards that can join projects and manage the research data management aspects of a project and can communicate this to project members.
#6 (26 points)	Stakeholders are not familiar with the steps that are needed to make a resource reusable by computers across multiple locations (Queralt et al [6]). Making data FAIR in a community without guides to make data FAIR is challenging.	See #1a for recommendations for step-by-step guides.
#7 (25 points)	Unclear who can or wants to handle data access requests especially after the project is stopped since funding is required to cover costs after the project is finished.	(Mental) health data are sensitive and will probably be available only upon a reasonable request. Therefore, someone needs to handle data access requests. The main problem of this barrier is the cost of someone that can handle the data access request after the project has ended. For funding recommendations, see #2.
#8 (25 points)	General Data Protection Regulation (GDPR) compliance needs to be embedded and the GDPR “right to be forgotten” should not be forgotten in the FAIR implementation plan (Overmars et al [29]).	Researchers need a clear guide on what the GDPR precisely entails. What is considered to be “personal data” can slightly differ between countries, indicating that there are still problems interpreting the GDPR. A (FAIR) data steward should be familiar with the GDPR and could provide help. See #1c. for FAIR data stewardship recommendations.
#9 (25 points)	Implementing the FAIR principles is a resource-intensive task, especially when carried out retrospectively. Resource costs associated with this task include the time, effort, and potentially standing up expenses (Alharbi et al [31]). FAIRifying data retrospectively remain challenging (Rocca-Serra and Sansone [43]).	For step-by-step guide and tooling recommendations see #1a. For funding recommendations, see #2.
#10 (25 points)	Legal challenges correspond to requirements that might pertain to the processing and sharing of the data (eg, accessibility rights and compliance with data protection regulations), both for meeting the “accessibility” and “reusability” criteria as well as for performing the FAIRification process itself (Wise et al 2022 [30]). The legal aspect of access rights is a significant issue due to its complexity and the lack of a clear process for accessing previously generated datasets (Alharbi et al 2021 [31]). Protecting patient data and privacy, which could require restrictions (also mentioned by Queralt-Rosinach et al [6]).	For legal challenges recommendations (eg GDPR), see #8.

a FAIR: Findable, Accessible, Interoperable, Reusable.

## Discussion

In this study, we identified 45 barriers that hamper the implementation of the FAIR principles in child and adolescent mental health research. Our study showed that most barriers are external barriers that have to do with tooling, and these mostly hamper all aspects of FAIR.

Although this research was conducted in the context of child and adolescent mental health, only 2 barriers were specific for mental health. One barrier is that mental-health data are collected in care modules that contain only questionnaires that made it difficult to represent in OMOP CDM and the other is that the *DSM* does not make use of identifiers. All other barriers were considered general barriers for making health data FAIR. The data in the child and adolescent mental health domain are therefore not more difficult to make FAIR than those of other health care domains. Moreover, the 2 mental health–specific barriers do not impact key components of the FAIR principles and are not in the top 10 barriers. One barrier would be solved by adding identifiers to the *DSM-5* (*Diagnostic and Statistical Manual of Mental Disorders* [Fifth Edition]) diagnoses. The other barrier is more complex as it requires community-wide guidelines on how to represent care modules (subcare pathways) in data standards.

This research was conducted in the Netherlands. However, the results show no indication of FAIR barriers that are country-specific. Meaning, that initiatives such as the United States Core Data for Interoperability Plus (USCDI+) [44] could learn from other initiatives across countries without taking country-specific barriers into account.

The top 10 barriers that we identified show that researchers struggle to put the FAIR principles into practice. Based on our study, we have three recommendations to enhance FAIR data use: (1) the structural use of FAIR data stewards [45], (2) creating clear step-by-step guides to make data FAIR, and (3) implementing sustainable funding systems to implement and maintain the FAIR data infrastructure.

Research from Garcia et al [5], Queralt-Rosinach et al [6], van Vlijmen et al [33], Jacobsen et al [35], and Mayer et al [46] already mentions challenges of implementing the FAIR principles. By combining all barriers, we intend to provide a valuable overview of barriers for researchers and funders and help them to be proactive in implementing structural solutions that can help prevent these barriers from occurring.

The results of this study show that many barriers exist in the data FAIRification process. As such, they provide guidance for researchers to prepare themselves before starting the FAIRification process by assessing our overview of barriers and implementing the recommendations. We decided to use the term “recommendations” instead of “strategies” because our recommendations can also apply to factors not directly influenced by researchers, such as organizational policies. We wanted to clearly convey this distinction. Furthermore, these results are intended to support researchers to better communicate their needs to organizations or grant providers for making their data FAIR. A caveat is that the recommendations could introduce new barriers. For instance, in our study, a FAIR data steward was closely connected to the consortium. He attended research meetings, was involved in solving data problems, and helped implementing the FAIR principles. However, the introduction of a FAIR data steward brings costs, which could be a financial barrier. Furthermore, there is a need for further capacity building of FAIR data stewards.

A strength of this study is that we used 3 distinct sources to identify FAIR data implementation barriers: an extensive list of barriers was created by combining the experience of 3 data managers with OMOP CDM, expert interviews, and literature. Another strength was the use of the framework by Cabana et al [23]; although the categorization of the barriers was originally designed to review physician guideline adherence, we successfully applied this to adherence to FAIR principles, a specific type of guideline, since the framework was also designed to standardize the reporting of barriers to adherence. The classification gives insight into the nature of the barriers, highlighting areas that could be improved within a FAIRification process.

A limitation of this study is that a bias may be present in the scoring of the barriers by the data managers. The data managers may have been inclined to give their self-experienced barriers a higher score. However, as the top 10 contain a mix of the 3 sources, expected bias seems limited. Bias could further have been introduced as the data managers had to score each barrier even if they had little to no knowledge regarding that barrier. This limitation is as much as possible mitigated by providing the data managers more explanation when necessary during the first round and the live meeting of the Delphi method. Another limitation is that we did not focus on facilitators in this study. However, the recommendations we provide make the FAIR data implementation process easier and could therefore be considered as facilitators. Finally, it could be argued that a small subset of data elements was used during the OMOP CDM implementation process, especially for the LDJ research site since they only had the PSQ as being mental health specific available in the chosen subset. However, we believe that the chosen subset was representative of the whole dataset to find all barriers. Even with introducing more data elements, it is likely that we would observe a repetition of already identified barriers instead of identifying new barriers. Moreover, not all barriers were related to data, some were technical, and focusing too much on the data aspect might have led to missing those technical barriers.

Future work should focus on designing a streamlined FAIR data process framework. This framework should contain practical and clear steps but should leave practical choices open to researchers or help them make informed decisions. Decision trees such as the one created by Verburg et al [36] could help researchers make their data FAIR. Their decision tree focuses on qualitative data; decision trees focused on other data or aspects of FAIR would be useful. The FAIR cookbook is a useful resource in this regard as well [42]. However, a FAIR recipe focused on making qualitative data FAIR is currently missing. The FAIR cookbook is written for expert users as it assumes familiarity with the FAIR principles, making it less of a starting point for beginners. Various communities have developed FAIR Implementation Profiles to provide guidance for implementing each principle [35,47,48], but a FAIR Implementation Profile focused on mental health data has not yet been developed, which would be relevant as further work.

In conclusion, 45 barriers that hamper the implementation of the FAIR principles were identified. The mental health domain does suffer largely from the same barriers to implementing the FAIR principles as other health care domains. We provided the top 10 barriers and recommendations for researchers to help them overcome these barriers. By providing this list, we hope that researchers are better prepared and motivated to start their FAIRification process and can better communicate their needs within their organization and towards grant providers. We strongly recommend the structural use of FAIR data stewards, creating clear step-by-step guides to make data FAIR, and the implementation of sustainable funding systems to maintain FAIR data infrastructure.

## Supplementary material

10.2196/59113 Multimedia Appendix 1 Justification of using the Delphi method according to the Guidance on Conducting and REporting DElphi Studies (CREDES) by Jünger et al [24].

10.2196/59113 Multimedia Appendix 2 All identified barriers and their type, affected FAIR principles, category, being specific to mental health, source, and total score. A forward slash in the source column indicates that the barrier was identified in multiple sources.

10.2196/59113 Multimedia Appendix 3 All notes, scores, and comments of each Delphi method round.

## Abbreviations

CDM: Common Data Model
CREDES: Guidance on Conducting and REporting DElphi Studies
DREAMS: Dutch REsearch in child and Adolescent Mental health
*DSM*: *Diagnostic and Statistical Manual of Mental Disorders*
*DSM-5*: *Diagnostic and Statistical Manual of Mental Disorders* (Fifth Edition)
ETL: Extract, Transform, Load
FAIR: Findable, Accessible, Interoperable, Reusable
GDPR: General Data Protection Regulation
LDJ: Dutch abbreviation for Learning Database Youth
OHDSI: Observational Health Data Sciences and Informatics
OMOP: Observational Medical Outcomes Partnership
PROMIS: patient-reported outcomes measurement information system
PSQ: Parenting Stress Questionnaire
USCDI+: United States Core Data for Interoperability Plus
