# Supplementary material for: Implementing Findable, Accessible, Interoperable, Reusable (FAIR) Principles in Child and Adolescent Mental Health Research: Mixed Methods Approach
Source: JMIR Ment Health. 2024 Dec 19;11:e59113. doi: 10.2196/59113 (PMC11683739; doi:10.2196/59113)
Supplement: Multimedia Appendix 1 [file mental-v11-e59113-s001.docx]

|  | **Description** | **Answer** |
| --- | --- | --- |
| **Rationale for the choice of the Delphi technique** |  |  |
| 1. Justification | The choice of the Delphi technique as a method of systematically collating expert consultation and building consensus needs to be well justified. When selecting the method to answer a particular research question, it is important to keep in mind its constructivist nature. | There are no units of measurement to determine barriers of implementing the FAIR principles. Measuring the consensus of implementers about our findings was deemed the best evidence we could provide. Therefore, we opted to use the Delphi method. |
| **Planning and design** |  |  |
| 2. Planning and process | The Delphi technique is a flexible method and can be adjusted to the respective research aims and purposes. Any modifications should be justified by a rationale and be applied systematically and rigorously. | We used a modified Delphi technique according the definition by Boulkedid et al. (2011). They define modified Delphi techniques as the combined use of a self-administered questionnaire and of a physical meeting of the experts to discuss the results or rate the indicators. |
| 3. Definition of consensus | Unless not reasonable due to the explorative nature of the study, an a priori criterion for consensus should be defined. This includes a clear and transparent guide for action on (a) how to proceed with certain items or topics in the next survey round, (b) the required threshold to terminate the Delphi process and (c) procedures to be followed when consensus is (not) reached after one or more iterations. | Since we use a 1-10 scale to rate how much the barriers hamper the FAIR data implementation, we agreed that a rating of three points or less difference between all three implementers is considered a consensus.  A. Barriers that have a rating that differs more than three points between any of the implementers are discussed during the online meeting.  B. The Delphi process is terminated in case one or more experts agree the Delphi method is not a suitable method.  C. A barrier will not be discussed if consensus is achieved. Otherwise the barrier is discussed during the online meeting to determine why there is no consensus. In case there is still no consensus, the barrier is discussed during a second live consensus meeting in which the research data manager will join the discussion to force a concensus. |
| **Study conduct** |  |  |
| 4. Informational input | All material provided to the expert panel at the outset of the project and throughout the Delphi process should be carefully reviewed and piloted in advance in order to examine the effect on experts’ judgements and to prevent bias. | The questionnaire is checked by three experts before sending it to the implementers. |
| 5. Prevention of bias | Researchers need to take measures to avoid directly or indirectly influencing the experts’ judgements. If one or more members of the research team have a conflict of interest, entrusting an independent researcher with the main coordination of the Delphi study is advisable. | Not applicable |
| 6. Interpretation and processing of results | Consensus does not necessarily imply the ‘correct’ answer or judgement; (non)consensus and stable disagreement provide informative insights and highlight differences in perspectives concerning the topic in question. | Insights and differences in perspectives emerging from the discussions are included in the results. |
| 7. External validation | It is recommended to have the final draft of the resulting guidance on best practice reviewed and approved by an external board or authority before publication and dissemination. | Final results are checked by the experts. |
| **Reporting** |  |  |
| 8. Purpose and rationale | The purpose of the study should be clearly defined and demonstrate the appropriateness of the use of the Delphi technique as a method to achieve the research aim. A rationale for the choice of the Delphi technique as the most suitable method needs to be provided. | The study aims to identify challenges of implementing the FAIR principles in child and adolescent mental health research. There are currently no units of measurement or guidelines to determine the barriers for implementing the FAIR principles. We argue that expert opinions are the best evidence we can provide, which makes the Delphi method a suitable method. |
| 9. Expert panel | Criteria for the selection of experts and transparent information on recruitment of the expert panel, socio-demographic details including information on expertise regarding the topic in question, (non)response and response rates over the ongoing iterations should be reported. | The implementers were chosen as they were involved in the project and experienced barriers to implementing the FAIR principles first hand during the implementation of OMOP CDM. |
| 10. Description of the methods | The methods employed need to be comprehensible; this includes information on preparatory steps (How was available evidence on the topic in question synthesised?), piloting of material and survey instruments, design of the survey instrument(s), the number and design of survey rounds, methods of data analysis, processing and synthesis of experts’ responses to inform the subsequent survey round and methodological decisions taken by the research team throughout the process. | This is described in the methods section of the paper. |
| 11. Procedure | Flow chart to illustrate the stages of the Delphi process, including a preparatory phase, the actual ‘Delphi rounds’, interim steps of data processing and analysis, and concluding steps. | See Figure 1. |
| 12. Definition and attainment of consensus | It needs to be comprehensible to the reader how consensus was achieved throughout the process, including strategies to deal with non-consensus. | This is described in the main text of the paper. |
| 13. Results | Reporting of results for each round separately is highly advisable in order to make the evolving of consensus over the rounds transparent. This includes figures showing the average group response, changes between rounds, as well as any modifications of the survey instrument such as deletion, addition or modification of survey items based on previous rounds. | Results for each round are reported separately in the Excel sheet. |
| 14. Discussion of limitations | Reporting should include a critical reflection of potential limitations and their impact of the resulting guidance. | Limitations are described in the discussion section of the main text. |
| 15. Adequacy of conclusions | The conclusions should adequately reflect the outcomes of the Delphi study with a view to the scope and applicability of the resulting practice guidance. | Conclusion is described in the main text of the paper. |
| 16. Publication and dissemination | The resulting guidance on good practice in palliative care should be clearly identifiable from the publication, including recommendations for transfer into practice and implementation. If the publication does not allow for a detailed presentation of either the resulting practice guidance or the methodological features of the applied Delphi technique, or both, reference to a more detailed presentation elsewhere should be made (e.g. availability of the full guideline from the authors or online; publication of a separate paper reporting on methodological details and particularities of the process (e.g. persistent disagreement and controversy on certain issues)). A dissemination plan should include endorsement of the guidance by professional associations and health care authorities to facilitate implementation. | Recommendations for transfer into practice and implementation are described in the main text of the paper. |
